# Techno-functional characterization of indigenous *Lactobacillus* isolates from the traditional fermented foods of Meghalaya, India

**DOI:** 10.1016/j.crfs.2020.01.002

**Published:** 2020-02-11

**Authors:** Sujit Das, Birendra Kumar Mishra, Subrota Hati

**Affiliations:** a Department of Rural Development and Agricultural Production, North- Eastern Hill University, Tura Campus, Tura, 794 001, Meghalaya, India; b Department of Dairy Microbiology, S.M.C College of Dairy Science, Anand Agricultural University, Anand, 388 110, Gujarat, India

**Keywords:** *Lactobacillus*, Probiotics, Fermentation, Fermented foods, Meghalaya

## Abstract

The rural tribal people of Meghalaya depend mostly on their ethnic fermented foods as a part of their regular diet and these fermented foods are considered to be a hub of healthy microorganisms. However, the efficacy of probiotic microorganisms is considered to be population-specific because of gut microflora variation in food habits and specific host-microbial interactions. Hence, a strong need for exploring novel indigenous microorganisms with rich probiotic potentiality is required. A few indigenous *Lactobacillus* isolates (from traditional fermented foods of Meghalaya) were studied extensively for its technological and probiotic attributes. The isolates could survive at pH 2–3 (*L. fermentum* K16 showed high cell count: pH 2–5.12 log CFU/ml; pH 3–5.76 log CFU/ml), against bile salts (*L. fermentum* K7 showed high cell count-5.36 log CFU/ml), gastric juices (pepsin and trypsin), and intestinal juice (pancreatin). The isolates showed α-galactosidase activity from 0.104-0.412 μM/ml and β-glucosidase activity ranging from 0.122-0.409 μM/ml. Exopolysaccharide production was in between 410 and 950 mg/L. Cell surface hydrophobicity was 71.57% (*L. rhamnosus* K4E) and auto-aggregation was 83% (*L. fermentum* K16) during the study. Highest proteolytic activity (0.671 nm) and cholesterol assimilation (52.57%) was exhibited by *L. fermentum* K16. The isolates showed high free radical scavenging activity by ABTS method up to 80.78% by isolate *L. fermentum* K7. Antibacterial activity and co-aggregation efficacy was also tested against *B. cereus, E. faecalis, S. dysenteriae, S. aureus, E. coli, L. monocytogenes, S. typhi.* These indigenous *Lactobacillus* isolates with high probiotic potentials could be exploited in the development of the traditional fermented foods of Meghalaya.

## Highlights

• Technological and probiotic attributes (*in vitro*) of eight indigenous *Lactobacillus* strains were studied.
• *Lactobacillus* strains were isolated from the traditional fermented foods of Meghalaya, India.
• *L. rhamnosus* K4E, *L. fermentum* K16, *L. fermentum* K7 and *L. plantarum* RD7 were considered predominant over the rest of the strains.
• The isolates could be employed for development of novel functional fermented foods.

## Graphical abstract

**Figure:**
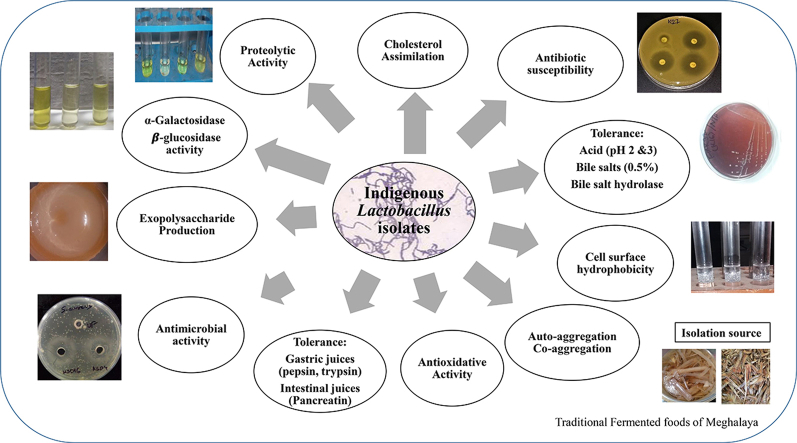

## 1 Introduction

Meghalaya (North-eastern region of India), is inhabited by various tribal population viz. Garo, Khasi and, Jaintia and the indigenous preparation and consumption of various fermented foods viz. fermented soybeans-*Tungrymbai*; fermented rice- *Wanti*; fermented rice beverages- *Chubitchi, Ka kiad*; fermented fish- *Tungtap, Nakham, Lungsiej*; fermented bamboo shoots- *Meakri*, are deeply rooted in them as part of their heritage and culture. The preparations of these traditional fermented food products are lesser-known since they remain restricted to individual households (Dewan and Tamang, 2007). Detailed studies on the probiotic potential and the bio-functional value of these products can provide valuable information and justify their potential use on a wider range.

A wider spectrum of lactic acid bacteria (LAB) have been isolated from various fermented foods across the globe and based upon their probiotic potentiality, they have been employed for novel foods and preparation in pharmaceutical industries (Monteagudo-Mera et al., 2012). Although LAB are usually considered as safe for consumption but to ensure further confirmation and as a criteria selection, *in vitro* serial tests have been developed as well as applied for identifying microorganisms with rich probiotic potentiality (Leahy et al., 2005). Furthermore, fermented foods containing LAB have been related to various probiotic characteristics such as improvising in lactose intolerance followed by digestion, reducing the level of cholesterol present in blood serum, suppressing cancerous cells, increased resistance to infections in the gastrointestinal and urogenital tracts (T Liong and Shah, 2005). For the production of traditional fermented foods and beverages as functional foods with high probiotic potentiality has been contributed significantly by the lactic acid bacteria present in it (Angmo et al., 2016).

The present study deals with the *Lactobacillus* isolates obtained from the indigenous fermented foods of Meghalaya were studied to determine their probiotic potentiality which may lead to the development of novel fermented foods further providing numerous health benefits to the people of Meghalaya and the other parts of India as well.

## 2 Materials and methods

### 2.1 Bacterial strains

A total of eight indigenous *Lactobacillus* strains (Table 1), isolated from the traditional fermented foods (fish- *Nakham*, curd, rice beverage- *Chubitchi*) of Meghalaya were selected for analysing their technological and probiotic properties, following a series of *in vitro* tests. These isolates were previously isolated and identified by Gram reaction, catalase test, sugar fermentation tests (API 50 CH kit). Molecular characterization of isolates was accomplished by 16s rRNA gene sequencing and the amplified gene sequences were submitted to NCBI (Mishra et al., 2017). Indicator strains for antimicrobial included *Bacillus cereus* ATCC 14459*, Enterococcus faecalis* NCDC 115*, Shigella dysenteriae* NCDC 107*, Staphylococcus aureus* MTCC 114*, Escherichia coli* ATCC 25922*, Listeria monocytogenes, Salmonella typhi* NCTC 5017*.* The test organisms were obtained from the culture collection maintained by Dept. of Dairy Microbiology, SMC College of Dairy Science, Anand Agricultural University, Anand, Gujarat, India.

**Table 1:** List of *Lactobacillus* isolates with NCBI GeneBank accession numbers.

Isolate code	Partially identified by BLAST	NCBI Genebank accession no.	Source (Traditional fermented foods of Meghalaya)
K3A	*Lactobacillus fermentum*	KU644575.1	Fermented fish *(Nakham)*
K7	*Lactobacillus fermentum*	KU213665.1	Curd sample
K16	*Lactobacillus fermentum*	KU213667.1	Fermented fish *(Nakham)*
K5	*Lactobacillus fermentum*	KU213668.1	Fermented fish *(Nakham)*
K4E	*Lactobacillus rhamnosus*	KX950834.1	Fermented Fish *(Nakham)*
K14	*Lactobacillus helveticus*	KU644578.1	Fermented fish *(Nakham)*
K27A	*Lactobacillus acidipiscis*	KY234394.1	Fermented fish *(Nakham)*
RD7	*Lactobacillus plantarum*	MF155569.1	Fermented Rice Beverage *(Chibitchi)*

### 2.2 Technological attributes

#### 2.2.1 Estimation of α-galactosidase and β-glucosidase activity

Crude enzyme extracts from the organisms were assayed for a-galactosidase activity according to the method of Scalabrini et al. (1998). The enzyme assay is based on the principle that when α-galactosidase enzyme acts on the substrate p-nitrophenyl-α-D-galactoside (HiMedia, India), a colorimetric reaction takes place which releases p-nitrophenol (pNP) in the medium. β-glucosidase activity was determined by measuring the rate of hydrolysis of p-nitrophenyl β-D-glucopyranoside (HiMedia, India) according to the method of Otieno and Shah (2007) and Scalabrini et al. (1998). The amount of p-nitrophenol released was measured spectrophotometrically using a UV–Vis spectrophotometer (Systronics, Ahmedabad) at 410 nm.

#### 2.2.2 Exopolysaccharide (EPS) production

For checking the EPS production, MRS broth was infused with sucrose (5% w/v) as the carbon source and at the rate of 2% lactic isolates were inoculated followed by incubation at 37 °C for 24 h. Cell pellets were removed by centrifugation at 14,000 rpm for 20 min and the cell-free supernatant was treated with 2.5% (v/v) of 80% (w/v) trichloroacetic acid (Merck). By further centrifugation at 15 000 g for 20 min, the precipitated proteins were removed. The resulting supernatant was treated with 3 vol of 95% chilled ethanol and incubated at 4 °C for 24 h for precipitating the EPS. The EPS was extracted by centrifugation at 14 000 rpm for 20 min. The samples were freeze-dried and weighed. The same procedure was performed on un-inoculated media and the weight of the resulting precipitate was subtracted from the amount of EPS produced by the LAB (Kimmel and F Roberts, 1998).

### 2.3 Probiotic attributes (*in vitro*)

#### 2.3.1 Acid tolerance

The survival rate was calculated as the number of colonies (log CFU/ml) that were enumerated on MRS agar medium after exposure to low pH conditions, 2.0, 3.0 and 7.0 in MRS broth at 37 °C at time intervals of 0, 1.5 and 3 h as compared to the initial cell concentration (Schillinger, 1989).

#### 2.3.2 Bile salt tolerance

The survival rate of each strain was expressed as number of viable cell colonies (log CFU/ml) that were enumerated on MRS agar medium after exposure to 0.5% (w/v) oxgall bile salts at time intervals of 0, 2 and 4 h in MRS broth at 37 °C as compared to that without bile salts (Schillinger, 1989).

#### 2.3.3 Resistance to simulated gastric fluid and intestinal fluid

The isolates were propagated in MRS broth overnight at 37 °C the cells were harvested by centrifugation at 12,000 rpm for 15 mins. The *Lactobacillus* cells (adjusted to10^8^ CFU/ml) were suspended in the artificial gastric juice (NaCl-0.73 g/L; KCl- 0.05 g/L; NaHCO_3_- g/L; pepsin- 0.3 g/L) with pH adjusted to 2.0 and 3.0 was incubated for 0, 2 and 4 h. Another gastric fluid was made with trypsin and was adjusted to the same pH conditions as mentioned above. The survival rate in terms of log CFU/ml was also checked by exposing the isolates to artificially made intestinal juice (0.1% w/v pancreatin and 0.3% w/v bile salts, pH 8.0) with incubation hours of 0, 2 and 4. Sterile saline solution (0.85% w/v NaCl) adjusted to pH 7.0 was used as control (Vidhyasagar and Jeevaratnam, 2013).

#### 2.3.4 Bile salt hydrolase activity

The *Lactobacillus* isolates were streaked on previously solidified MRS agar plates containing 0.5% (w/v) bile, sodium taurodeoxycholate hydrate, sodium taurocholate (Sigma) and 0.37 g/L of CaCl_2_ followed by 48 h incubation at 37 °C anaerobically in a Gaspak jar. The activity was indicated by precipitation around the streak (Kathiriya et al., 2018).

#### 2.3.5 Proteolytic activity

The peptides released by the *Lactobacillus* isolates in soymilk medium were measured as absorbance of free amino acids at 340 nm by using Double beam Spectrophotometer 2202 S, Systronics Ltd., India following the o-phthaldialdehyde (OPA) method of Donkor et al. (2007).

#### 2.3.6 Antibiotic susceptibility

Antibiotic resistance of the *Lactobacillus* isolates was determined on MRS agar by the disk diffusion method (Clinical and Laboratory Standards Institute (CLSI), 2011). The following antimicrobial agents viz. inhibitors of cell wall synthesis-azithromycin (AZM; 15 μg), ampicillin (A; 10 μg), vancomycin (VA; 30 μg), methicillin (MET; 15 μg), oxacillin (OX; 1 μg) inhibitor of nucleic acid synthesis- norfloxacin (NX; 10 μg); inhibitor of protein synthesis-kanamycin (K; 30 μg), streptomycin (S; 10 μg), erythromycin (E; 15 μg), tetracycline (TE; 30 μg) have been tested.

#### 2.3.7 Antimicrobial activity

The agar well diffusion assay was used to study the antimicrobial activity of the selected *Lactobacillus* strains (Schillinger, 1989). All the isolates were evaluated for antimicrobial activity against ten major test organisms i.e. *B. cereus, E. faecalis, S. dysenteriae, S. aureus, E. coli, L. monocytogenes, S. typhi*.

#### 2.3.8 Antioxidative activity

This antioxidative assay was based on the capability of the *Lactobacillus* isolates to scavenge 2, 2′- azino-bis (ethylbenzthiazoline-6-sulfonic acid (ABTS) radical cation as stated by Emad et al. (Emad and Sanaa, 2012). By determining the decrease in absorbance at different concentrations, the antioxidative activity of the tested samples was calculated by using the following equation:

(1) E=[(Ac-At)/Ac]×100

where, At = absorbance of tested samples and Ac = the absorbance of ABTS radical respectively.

#### 2.3.9 Cholesterol assimilation

The method of Walker and Gilliland (1993) was adopted for checking the assimilation of cholesterol by the *Lactobacillus* isolates used in this study. The results were recorded as cholesterol reducing percentage in supernatant broth (test) as compared to the un-inoculated broth (blank).

(2) % cholesterol assimilation=[(C0-CI)/CO]x100

where, C_O_: OD_550nm_ of MRS broth supernatant with culture, C_I_: OD_550nm_ of MRS broth supernatant without culture.

#### 2.3.10 Cell surface hydrophobicity

The bacterial adhesion to hydrocarbons was determined by following the method of Rosenberg et al. (1980). The surface hydrophobicity (%) was calculated as the percent decrease in the absorbance of the aqueous phase (A1) after mixing and phase separations relative to that of original suspension (A0) as:

(3) $$\text{\% H}\;=\;\frac{A0-A1}{A0}\;\text{×}\;100$$

#### 2.3.11 Cellular aggregation

Aggregation study was examined for the eight selected effective *Lactobacillus* spp. from the ethnic fermented foods based on their sedimentation characteristics (Schillinger, 1989). The percent difference between the initial and final absorbance would give an index of cellular auto-aggregation that can be expressed as follows:

(4) Agg. % = 100 X (A_initial_-A_final_)/Ab_initial_

where, A_initial_ = initial absorbance at 600 nm; A_final_ = final absorbance at 600 nm; Agg% = Aggregation index.

#### 2.3.12 Co-aggregation assay

An equal volume of cells of the different *Lactobacillus* spp. and test organisms viz. *Bacillus cereus* (ATCC 14459), *Enterococcus faecalis* (NCDC 115)*, Staphylococcus aureus* (MTCC 114)*, Escherichia coli* (ATCC 25922) *Salmonella typhi* (NCTC 5017) and *Enterococcus faecalis* (NCDC 115) (1:1 v/v) were mixed and incubated at 37 °C without agitation as per the method of Ekmekci et al. (2009) with few modifications. Absorbance (A_600nm_) of the mixtures as stated above were supervised during various incubation hours (0 h, 4 h, and 24 h) with co-aggregation percentage expressed as:

(5) Co-aggregation (%) = [(A_pathogen_ + A_*Lactobacillus*_)/2-A_mix_ (A_pathogen_+ A_*Lactobacillus*_)/2] x 100

where, A_pathogen_, A_*Lactobacillus*_ and A_mix_ represents the absorbance at 600 nm of the individual pathogen, *Lactobacillus* spp. and their mixture after incubation for 0 h, 4 h and 24 h respectfully.

### 2.4 Statistical analysis

Data presented in the study are an average of three independent assays and the results obtained were expressed as mean ± standard deviation (M±SD). One way analysis of variance (ANOVA) was applied and comparison was made through Bonferroni's test with the least significant difference of p ≤ 0.05 using the IBM SPSS Statistical Program Ver. 20.

## 3 Results

### 3.1 α-galactosidase and β-glucosidase activity

**Fig. 1:**
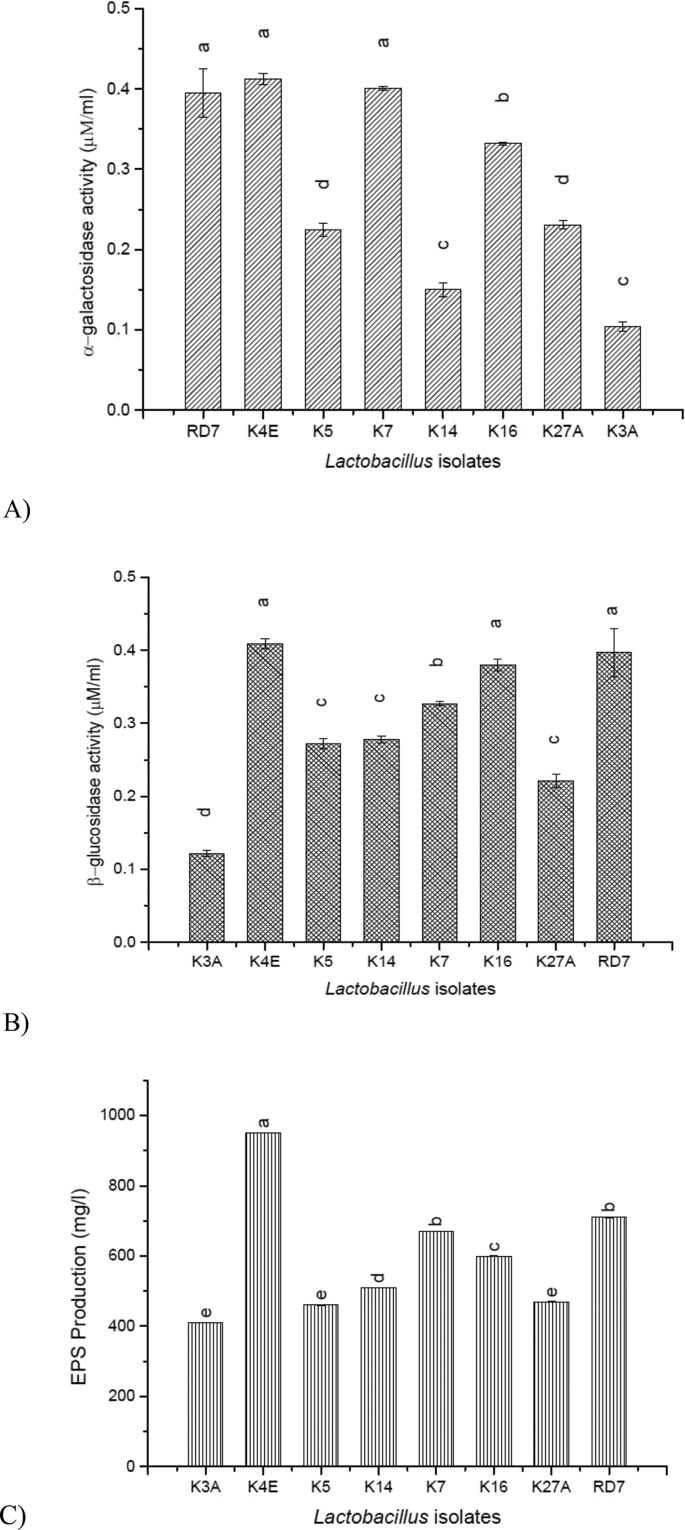
Technological attributes of the *Lactobacillus* isolates. (A) α-galactosidase and (B) β-glucosidase activity of the indigenous *Lactobacillus* isolates in soymilk medium; (C) Exopolysaccharide (EPS) production. Values are mean ± standard deviation of triplicate determinations (n = 3). Values bearing different superscripts differ significantly (P < 0.05).

The α-galactosidase activity of the *Lactobacillus* isolates was studied in soy milk medium which differed significantly (P < 0.05) amongst one another. The time of fermentation time was a primary factor for α-galactosidase activity during the 24 h incubation at 37 °C. Out of all the isolates, the highest release of α-galactosidase activity was reported in K4E (0.412 ± 0.0072 μM/ml) followed by K7 (0.401 ± 0.0025 μM/ml), RD7 (0.395 ± 0.03 μM/ml) and K16 (0.332 ± 0.0019 μM/ml) as presented in Fig. 1(A). The indigenous *Lactobacillus* strains used in this study exhibited different levels of β-glucosidase activity during their growth under optimal conditions in soymilk. Analysis of variance showed that the response presented by K4E (0.409 ± 0.007 μM/ml) followed by RD7 (0.397 ± 0.033 μM/ml), K16 (0.380 ± 0.008 μM/ml) and K7 (0.327 ± 0.002 μM/ml) was significantly higher (P < 0.05) than that of the rest of strains Fig. 1(B).

### 3.2 EPS quantification

In the present study, all of the *Lactobacillus* isolates produced on an average 500 mg of EPS per liter (dry mass basis) in a semi-defined medium. Comparatively with the rest of the cultures, *L. rhamnosus* K4E reported with highest EPS production with 950 ± 0.256 mg/L followed by *L. plantarum* RD7 (710 ± 0.388 mg/L), K7 (670 ± 0.185 mg/L) and K16 (600 ± 0.367 mg/L) respectively Fig. 1(C).

### 3.3 Tolerance to acid, bile, gastric and intestinal juices

**Fig. 2:**
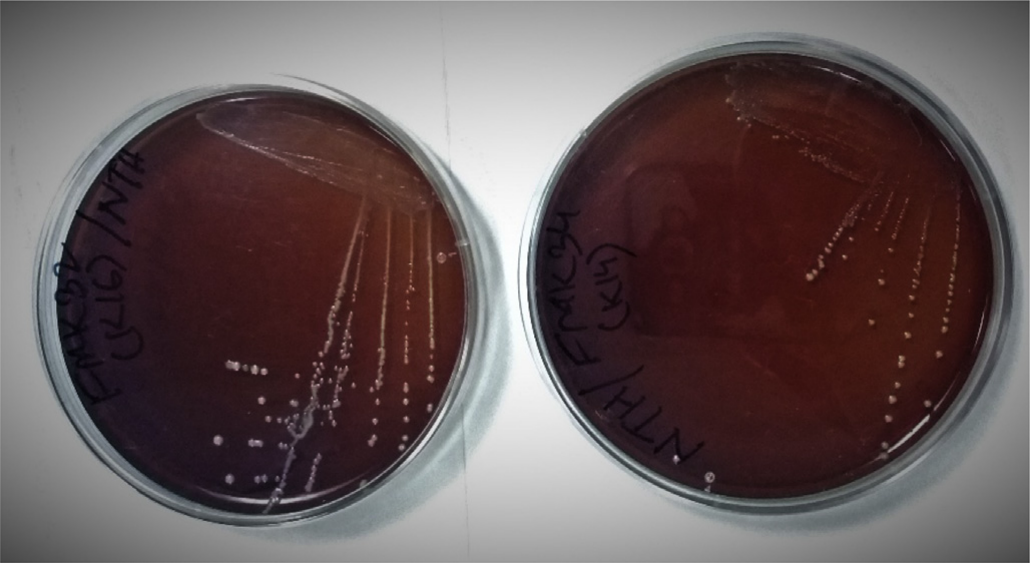
Bile salt hydrolase activity of the indigenous *Lactobacillus* isolates.

From the results depicted in Table 2, it can be interpreted that the growth of the strains was suppressed at lower pH after 2 h incubation in terms of viable cell count followed by more gradual cell count reduction after 4 h of incubation. The indigenous *Lactobacillus* isolates were relatively more resistant at pH 3 as compared to pH 2 conditions. Comparatively, isolate K16 showed higher viable cell count in pH 2 (5.12 ± 0.026 log CFU/ml) and pH 3 (5.76 ± 0.06 log CFU/ml) conditions after 4 h incubation. It was also observed at control pH (7.0) used in the study, the growth was relatively higher than pH 2 and pH 3 for all the eight isolates. Similarly, the eight lactic isolates were able to tolerate 0.5% bile concentration at 37 °C and the gradual reduction in viable cells after 3 h was noticed as depicted in Table 3 and the cell growth in the control at pH 7.0 was quite higher than that. Comparatively, K7 showed a higher cell count of 5.36 ± 0.025 log CFU/ml after 4 h incubation. All of the eight isolates used in this study showed a positive bile salt hydrolase activity which was achieved by the growth of opaque colonies (Fig. 2), presumably resulting in bile salts deconjugation.

**Table 2:** Viable cell counts (log CFU/ml) of *Lactobacillus* isolates in different pH conditions.

Strains	2.0 pH			3.0 pH			Control (pH 7.0)
	0 h	2 h	4 h	0 h	2 h	4 h	
K3A	7.47 ± 0.018^a^	5.20 ± 0.022^d^	4.17 ± 0.075^e^	8.14 ± 0.075^b^	5.85 ± 0.038^d^	5.03 ± 0.135^d^	8.29 ± 0.085^b^
K4E	8.83 ± 0.010^b^	5.15 ± 0.011^d^	4.65 ± 0.028^e^	8.50 ± 0.041^b^	5.82 ± 0.035^d^	5.26 ± 0.01^d^	9.73 ± 0.054^c^
K5	7.61 ± 0.015^a^	5.02 ± 0.030^d^	4.62 ± 0.05^e^	8.82 ± 0.050^b^	5.32 ± 0.074^d^	4.73 ± 0.045^e^	9.15 ± 0.025^c^
K7	7.69 ± 0.018^a^	5.42 ± 0.075^d^	4.78 ± 0.015^e^	8.57 ± 0.030^b^	6.67 ± 0.075^f^	5.15 ± 0.01^d^	9.75 ± 0.02^c^
K14	8.89 ± 0.05^b^	5.59 ± 0.010^d^	4.45 ± 0.054^e^	8.35 ± 0.054^b^	6.91 ± 0.060^f^	5.44 ± 0.025^d^	8.10 ± 0.11^b^
K16	8.29 ± 0.04^b^	5.85 ± 0.028^d^	5.12 ± 0.026^d^	8.68 ± 0.040^b^	6.15 ± 0.054^f^	5.76 ± 0.06^d^	9.54 ± 0.085^c^
K27A	7.43 ± 0.05^a^	5.79 ± 0.028^d^	4.30 ± 0.030^e^	8.78 ± 0.045^b^	5.20 ± 0.021^d^	5.57 ± 0.045^d^	8.87 ± 0.08^c^
RD7	7.11 ± 0.06^a^	5.74 ± 0.030^d^	4.80 ± 0.12^e^	8.26 ± 0.026^b^	6.19 ± 0.030^f^	5.69 ± 0.06^d^	9.67 ± 0.07^b^

Values are mean ± SD of three independent determinations (n = 3) of each sample. Values bearing different superscripts in each column differ significantly (P < 0.05).

**Table 3:** Viable cell counts (log CFU/ml) of isolates in bile salts (0.5%) at different incubation hours.

Strains	0 h	2 h	4 h	Control
K3A	6.45 ± 0.025^a^	5.28 ± 0.05^c^	4.45 ± 0.10^d^	7.25 ± 0.08^b^
K4E	7.26 ± 0.06^b^	6.16 ± 0.09^a^	5.12 ± 0.025^c^	7.33 ± 0.09^b^
K5	6.74 ± 0.010^a^	5.53 ± 0.030^c^	4.48 ± 0.025^d^	7.18 ± 0.032^b^
K7	7.12 ± 0.05^b^	6.35 ± 0.09^a^	5.36 ± 0.025^c^	7.45 ± 0.030^b^
K14	7.33 ± 0.08^b^	6.70 ± 0.07^a^	4.27 ± 0.01^d^	7.42 ± 0.08^b^
K16	7.27 ± 0.08^b^	6.72 ± 0.08^a^	5.23 ± 0.06^c^	7.65 ± 0.025^b^
K27A	6.49 ± 0.027^a^	5.13 ± 0.028^c^	4.12 ± 0.03^d^	7.01 ± 0.030^b^
RD7	7.04 ± 0.030^b^	5.87 ± 0.024^c^	4.88 ± 0.01^d^	7.20 ± 0.05^b^

Values are mean ± SD of three independent determinations (n = 3) of each sample. Values bearing different superscripts in each column differ significantly (P < 0.05).

The gastrointestinal transit tolerance of the eight *Lactobacillus* isolates was determined by exposing the cell suspensions to simulated gastric juice containing pepsin and trypsin followed by artificial intestinal juice containing pancreatin. In correlation to the results of pH tolerance stated above, all the indigenous *Lactobacillus* isolates were relatively more resistant at pH 3 as compared to pH 2 conditions in the case of both the simulated gastric juices viz. trypsin and pepsin. After 4 h of incubation, isolate RD7 showed cell count of 5.45 ± 0.04 log CFU/ml in and 5.51 ± 0.04 log CFU/ml in pH 2 and pH 3 conditions in gastric juice with trypsin supplementation (Table 4a). K4E (in pepsin) showed the highest number of viable cell counts of 5.12 ± 0.14 log CFU/ml and 5.30 ± 0.05 log CFU/ml in pH 2 and pH 3 conditions in gastric juice with pepsin supplementation (Table 4b). Similarly, on exposure to simulated intestinal fluid (pancreatin), all the isolates showed resistance after 4 h incubation with a highest cell count of isolate K4E (6.917 ± 0.15 log CFU/ml) respectively (Table 5). Hence, from the above results, it can state that the *Lactobacillus* isolates were adapted to grow significantly in both acidic and neutral environments.

**Table 4a:** Survivability of lactobacilli isolates in simulated gastric juice (Trypsin).

Isolates	Viable cell counts (log CFU/ml) at different incubation hours								
	0 h	2 h	4 h	0 h	2 h	4 h	0 h	2 h	4 h
pH 2 (Trypsin)				pH 3 (Trypsin)			Control (pH 7)		
K3A	7.02 ± 0.03^a^	5.12 ± 0.02	3.12 ± 0.50^e^	8.55 ± 0.15^f^	6.22 ± 0.16^b^	4.01 ± 0.11^c^	7.45 ± 0.12^a^	7.61 ± 0.07^a^	8.15 ± 0.03^f^
K4E	7.20 ± 0.05^a^	6.54 ± 0.21^b^	5.02 ± 0.13^c^	8.16 ± 0.05^f^	7.15 ± 0.08^a^	5.25 ± 0.04^c^	8.55 ± 0.08^f^	8.82 ± 0.05^f^	9.14 ± 0.02^g^
K5	7.12 ± 0.07^a^	6.78 ± 0.07^b^	4.48 ± 0.06^d^	8.65 ± 0.10^f^	6.33 ± 0.04^b^	5.07 ± 0.02^c^	8.22 ± 0.05^f^	8.67 ± 0.12^f^	8.99 ± 0.03^f^
K7	7.12 ± 0.12^a^	6.36 ± 0.02^b^	4.65 ± 0.08^d^	8.45 ± 0.15^f^	7.01 ± 0.05^a^	5.17 ± 0.11^c^	8.33 ± 0.03^f^	8.99 ± 0.06^f^	9.15 ± 0.05^g^
K14	7.20 ± 0.06^a^	5.22 ± 0.11^c^	3.10 ± 0.02^e^	8.55 ± 0.09^f^	6.25 ± 0.06^b^	4.38 ± 0.02^c^	8.45 ± 0.18^f^	8.85 ± 0.06^f^	9.08 ± 0.11^g^
K16	7.17 ± 0.02^a^	6.59 ± 0.11^b^	4.91 ± 0.03^d^	8.21 ± 0.10^f^	7.00 ± 0.09^a^	5.22 ± 0.02^c^	8.20 ± 0.02^f^	8.51 ± 0.02^f^	9.13 ± 0.18^g^
K27A	7.16 ± 0.07^a^	5.41 ± 0.01^c^	3.65 ± 0.07^e^	8.11 ± 0.08^f^	6.45 ± 0.21^b^	4.00 ± 0.02^c^	8.25 ± 0.04^f^	8.73 ± 0.05^f^	9.03 ± 0.09^g^
RD7	7.40 ± 0.10^a^	6.01 ± 0.08^b^	5.45 ± 0.04^c^	8.19 ± 0.09^f^	7.02 ± 0.12^a^	5.51 ± 0.02^c^	8.56 ± 0.07^f^	9.10 ± 0.02^g^	9.31 ± 0.08^g^

**Table 4b:** Survivability of lactobacilli isolates in simulated gastric juice (Pepsin).

Isolates	Viable cell counts (log CFU/ml) at different incubation hours								
	0 h	2 h	4 h	0 h	2 h	4 h	0 h	2 h	4 h
pH 2 (Pepsin)				pH 3 (Pepsin)			Control (pH 7)		
K3A	8.10 ± 0.09^b^	6.41 ± 0.01^c^	3.25 ± 0.08^f^	8.11 ± 0.02^b^	7.20 ± 0.08^a^	4.97 ± 0.12^d^	8.00 ± 0.07^b^	8.40 ± 0.02^b^	8.88 ± 0.18^b^
K4E	8.11 ± 0.07^b^	6.11 ± 0.09^c^	5.12 ± 0.14^d^	8.23 ± 0.08^b^	7.32 ± 0.11^a^	5.30 ± 0.05^d^	8.35 ± 0.08^b^	8.82 ± 0.06^b^	9.22 ± 0.12^g^
K5	7.33 ± 0.05^a^	5.76 ± 0.17^d^	3.42 ± 0.08^f^	7.82 ± 0.11^a^	6.13 ± 0.07^c^	4.55 ± 0.22^e^	8.11 ± 0.17^b^	8.38 ± 0.15^b^	8.90 ± 0.06^b^
K7	8.15 ± 0.10^b^	6.92 ± 0.18^c^	4.85 ± 0.09^e^	8.45 ± 0.15^b^	6.23 ± 0.08^c^	5.17 ± 0.13^e^	8.23 ± 0.06^b^	8.59 ± 0.06^b^	8.95 ± 0.09^b^
K14	7.71 ± 0.13^a^	6.02 ± 0.04^c^	4.12 ± 0.11^e^	8.01 ± 0.07^b^	6.11 ± 0.06^c^	4.08 ± 0.03^e^	8.22 ± 0.09^b^	8.41 ± 0.02^b^	8.77 ± 0.14^b^
K16	8.07 ± 0.05^b^	6.55 ± 0.30^c^	4.51 ± 0.06^e^	8.15 ± 0.18^b^	6.42 ± 0.16^c^	5.10 ± 0.02^d^	8.53 ± 0.16^b^	8.81 ± 0.016^b^	9.11 ± 0.22^g^
K27A	7.91 ± 0.16^a^	5.11 ± 0.14^d^	3.02 ± 0.04^f^	8.21 ± 0.07^b^	6.22 ± 0.14^c^	4.12 ± 0.20^e^	8.35 ± 0.14^b^	8.68 ± 0.07^b^	8.93 ± 0.09^b^
RD7	8.10 ± 0.15^b^	6.16 ± 0.21^c^	4.34 ± 0.07^e^	8.57 ± 0.12^b^	6.42 ± 0.20^c^	4.91 ± 0.06^d^	8.45 ± 0.12^b^	8.81 ± 0.09^b^	9.05 ± 0.12^g^

Values are mean ± SD of three independent determinations (n = 3) of each sample. Values bearing different superscripts in each column differ significantly (P < 0.05).

**Table 5:** Survivability of lactobacilli isolates in simulated intestinal juice (Pancreatin).

Isolates	Viable cell counts (log CFU/ml) at different incubation hours					
	0 h	2 h	4 h	0 h	2 h	4 h
Intestinal juices (pH 8)				Control (pH 7)		
K3A	7.196 ± 0.05^a^	6.812 ± 0.06^c^	5.744 ± 0.10^d^	8.326 ± 0.19^b^	8.412 ± 0.14^b^	8.601 ± 0.15^b^
K4E	8.750 ± 0.10^b^	7.720 ± 0.21^a^	6.917 ± 0.15^c^	9.778 ± 0.24^e^	9.802 ± 0.20^e^	9.911 ± 0.25^e^
K5	7.525 ± 0.09^a^	6.646 ± 0.16^c^	5.145 ± 0.18^d^	8.735 ± 0.10^b^	8.880 ± 0.30^b^	9.075 ± 0.18^e^
K7	8.733 ± 0.08^b^	7.505 ± 0.30^a^	6.106 ± 0.30^c^	9.662 ± 0.15^e^	9.720 ± 0.18^e^	9.781 ± 0.07^e^
K14	7.812 ± 0.15^a^	6.623 ± 0.33^c^	5.417 ± 0.08^d^	8.678 ± 0.09^b^	8.875 ± 0.18^b^	8.901 ± 0.15^b^
K16	8.411 ± 0.20^b^	7.112 ± 0.07^a^	6.885 ± 0.015^c^	9.522 ± 0.04^e^	9.621 ± 0.07^e^	9.726 ± 0.30^e^
K27A	7.965 ± 0.08^a^	6.271 ± 0.09^c^	5.525 ± 0.07^d^	8.012 ± 0.16^b^	8.112 ± 0.15^b^	8.267 ± 0.33^b^
RD7	8.225 ± 0.04^b^	7.112 ± 0.10^a^	6.878 ± 0.05^c^	9.335 ± 0.20^e^	9.458 ± 0.10^e^	9.600 ± 0.24^e^

Values are mean ± SD of three independent determinations (n = 3) of each sample. Values bearing different superscripts in each column differ significantly (P < 0.05).

### 3.4 Proteolytic activity

**Fig. 3:**
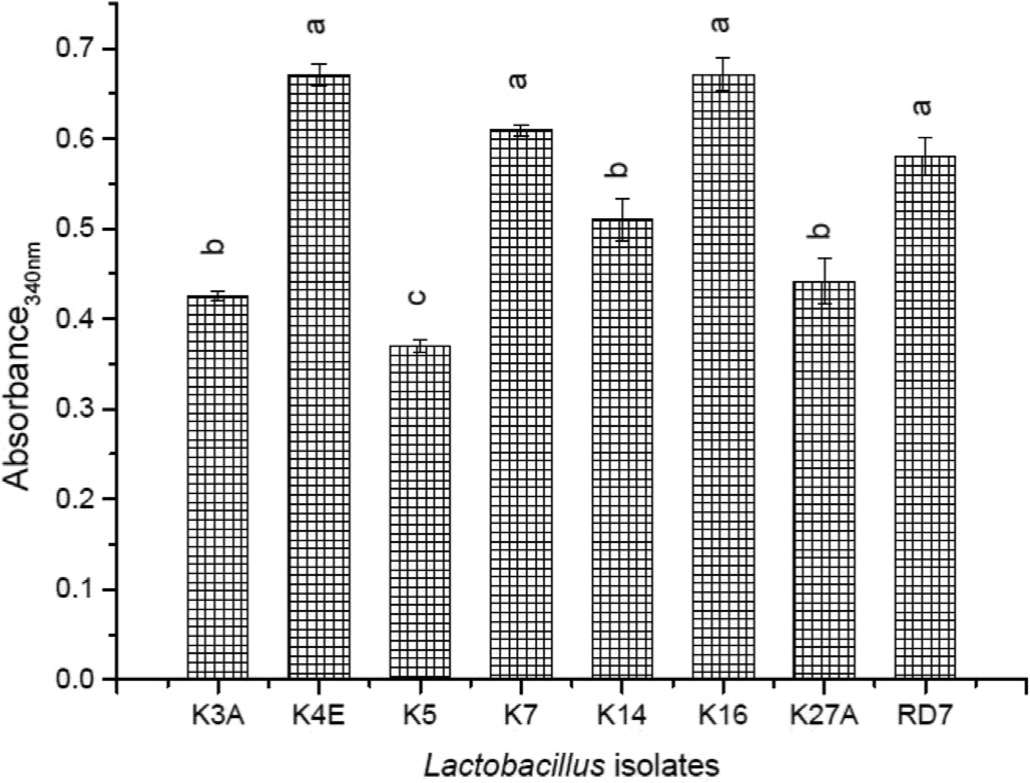
Proteolytic activity of the indigenous *Lactobacillus* isolates.

Through hydrolysis of peptides in the soymilk medium, amino acids might be liberated by the *Lactobacillus* isolates that were used in this study. The activity ranged from 0.370 to 0.671 nm (Fig. 3). The highest absorbance was claimed by isolate K16 (0.671) followed by K4E (0.670) and K7 (0.609). The lowest was reported by isolate K5 (0.370). The extent of proteolysis varied among *Lactobacillus* strains and appeared to be time dependent.

### 3.5 Antibiotic susceptibility

**Fig. 4:**
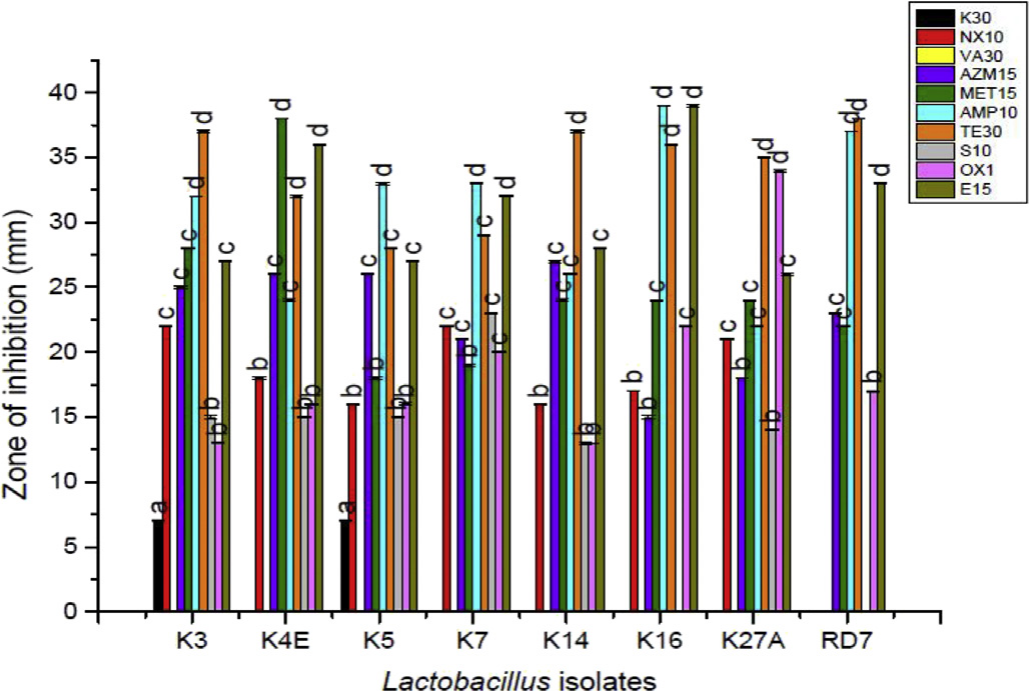
Antibiotic susceptibility of the indigenous *Lactobacillus* isolates.

The variable susceptibility of *Lactobacillus* isolates to the ten clinically important antibiotics was observed by Kirby–Bauer disc diffusion method as recommended by the Clinical and Laboratory Standards Institute (Clinical and Laboratory Standards Institute (CLSI), 2011). All of the strains showed resistance to vancomycin and lower resistance was observed in K3A (7 mm) and K5 (7 mm) but the rest of the isolates proved to be resistant to kanamycin. RD7 proved to be resistant to norfloxacin and K16 and RD7 showed resistance to streptomycin. The diameter (mm) of the zone of inhibition around the antibiotic discs was measured using an antibiotic zone scale for each of the *Lactobacillus* isolates to check the susceptibility, respectively in Fig. 4.

### 3.6 Antimicrobial activity

**Fig. 5:**
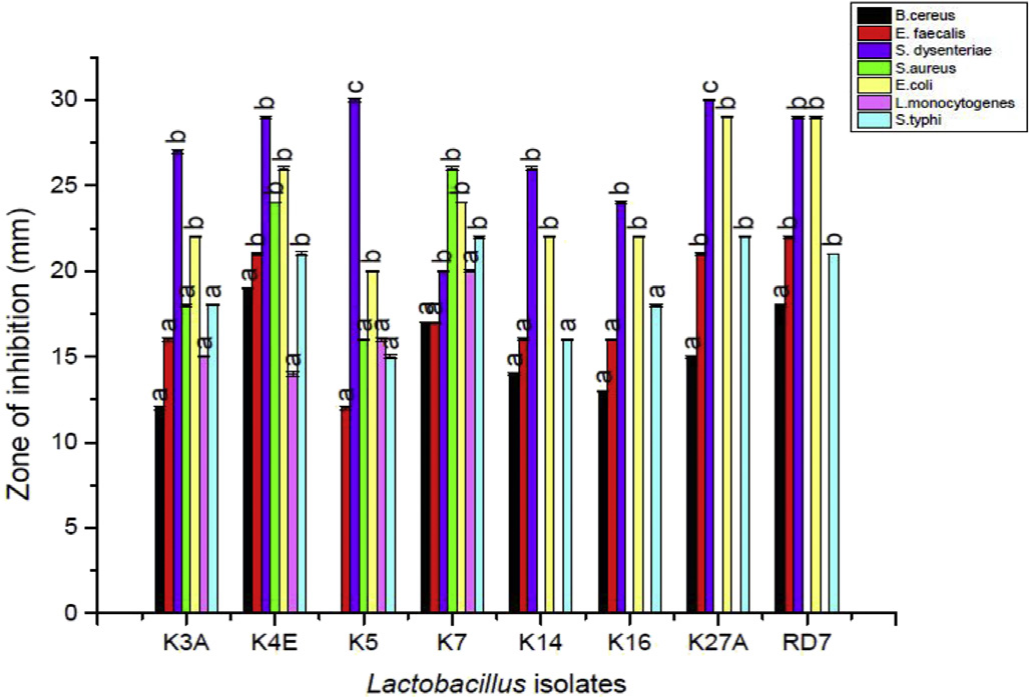
Antimicrobial activity of the indigenous *Lactobacillus* isolates.

The agar well diffusion method was employed to study the antimicrobial activity of *Lactobacillus* isolates against seven test organisms as shown in Fig. 5. The zones of inhibition of the indicator organisms were analysed by measuring the zone of inhibition that ranged from 14 to 30 mm in diameter. In the case of K27A, *S. dysenteriae* was considered as the most sensitive with a 30 mm diameter of ZOI followed by K4E, RD7 against *S. dysenteriae* (29 mm) and K27A, RD7 against *E. coli* (29 mm). There was no reports of antimicrobial activity by strains K14, K16 K27A and RD7 against *S. aureus* and *L. monocytogenes.*

### 3.7 Antioxidative activity

**Fig. 6:**
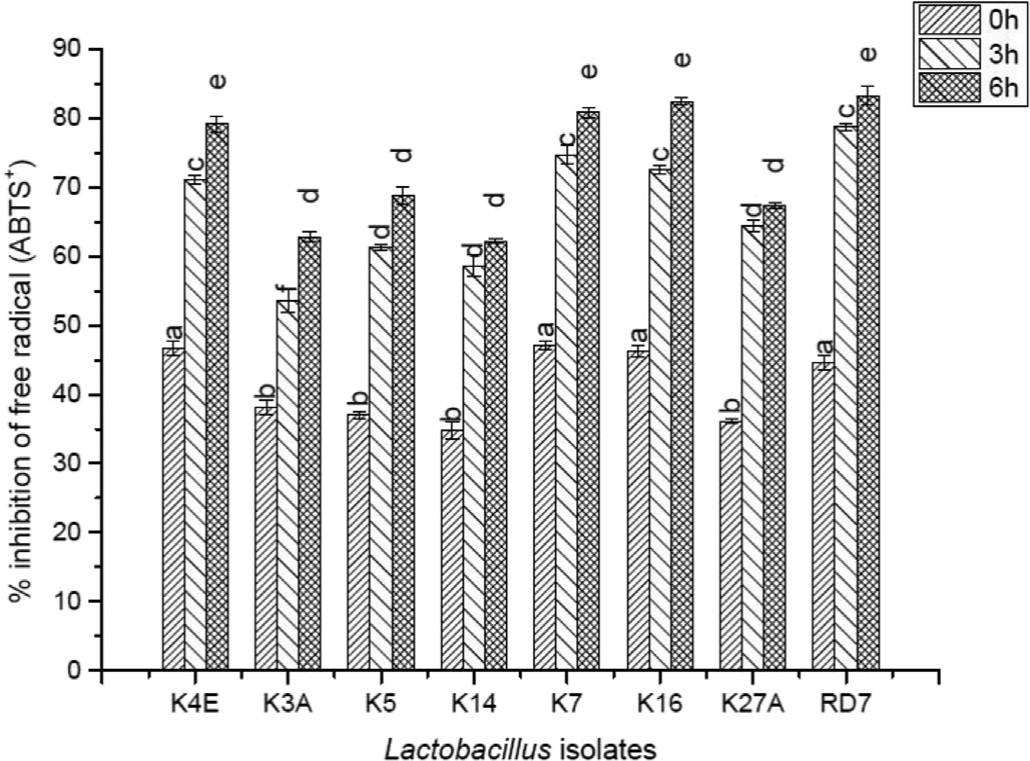
Antioxidative activity of the indigenous *Lactobacillus* isolates.

By ABTS^+^ scavenging method, antioxidative efficacy of eight indigenous *Lactobacillus* cultures was evaluated as represented in Fig. 6. The extent of inhibition was noticed to be increased significantly amongst these isolates after a period of 0, 3 and 6 h. K7 had provided the highest scavenging percentage viz. 47.14 ± 0.62, 74.71 ± 1.44 and 80.78 ± 0.78 after the above-stated incubation hours as compared to other isolates.

### 3.8 Cholesterol assimilation

**Fig. 7:**
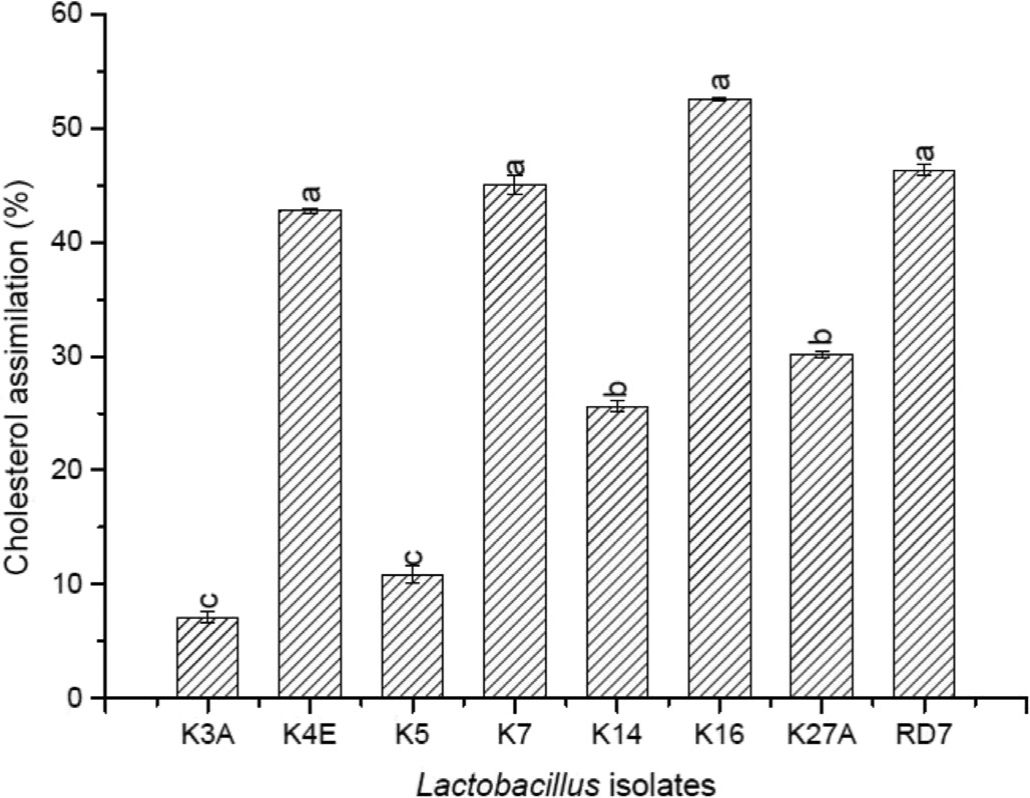
Cholesterol assimilation of the indigenous *Lactobacillus* isolates.

The capability of the eight indigenous *Lactobacillus* strains to assimilate cholesterol in MRS media was determined. All the eight strains investigated were successful at assimilating cholesterol following 24 h of incubation in cholesterol-containing MRS, as seen in Figure. In this study as shown in Fig. 7, the percentage reduction in cholesterol was significantly (P < 0.05) higher in K16 (52.57 ± 0.11) followed by RD7 (46.33 ± 0.47) and K5 (42.74 ± 0.22).

### 3.9 Cell surface hydrophobicity

**Fig. 8:**
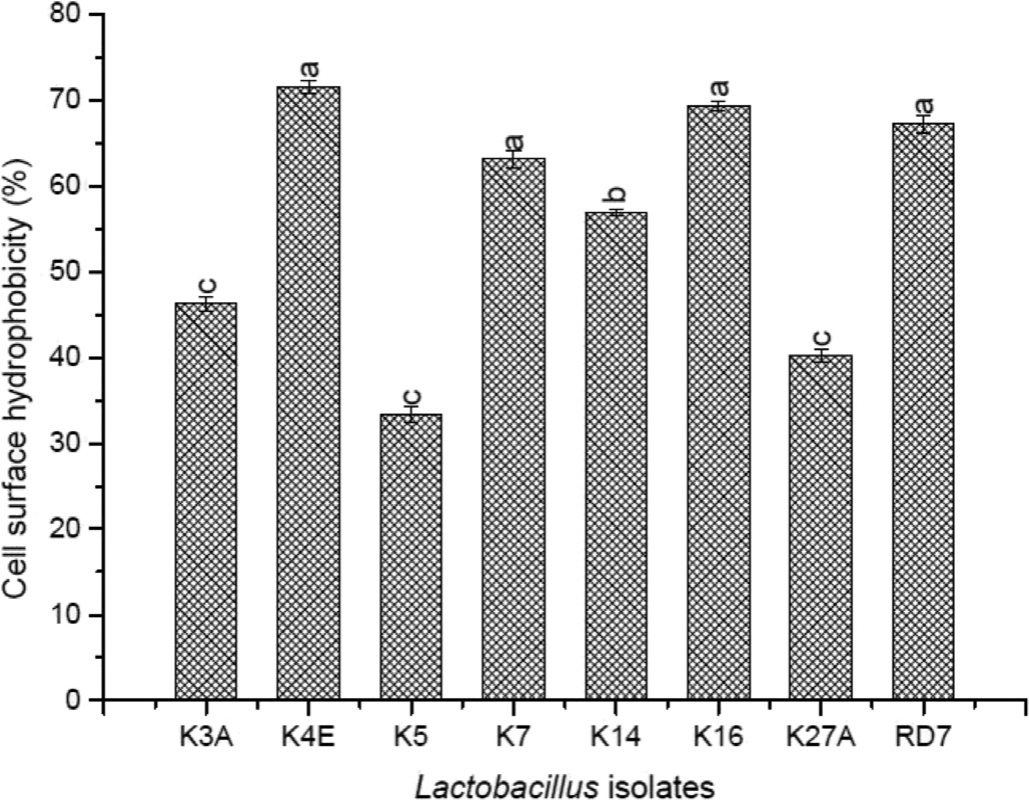
Cell Surface Hydrophobicity of the indigenous *Lactobacillus* isolates.

Microbial adhesion to hydrocarbon (MATH) or Cell-surface hydrophobicity method was employed based on the transfer of microbial cells into the hexadecane phase. The results were expressed as a percentage of the cell population which had passed into the hydrophobic phase of the solvent (Fig. 8). K4E was found to be more adherent (71.57 ± 0.7) to n-hexadecane followed by K16 (69.30 ± 0.056), RD7 (67.21 ± 1.05) and K7 (63.10 ± 1.05). However, there were no significant differences among the rest of the strains. Besides, K5 had the lowest adherence (33.40 ± 0.95) efficacy amongst all.

### 3.10 Cellular aggregation and Co-aggregation

**Fig. 9:**
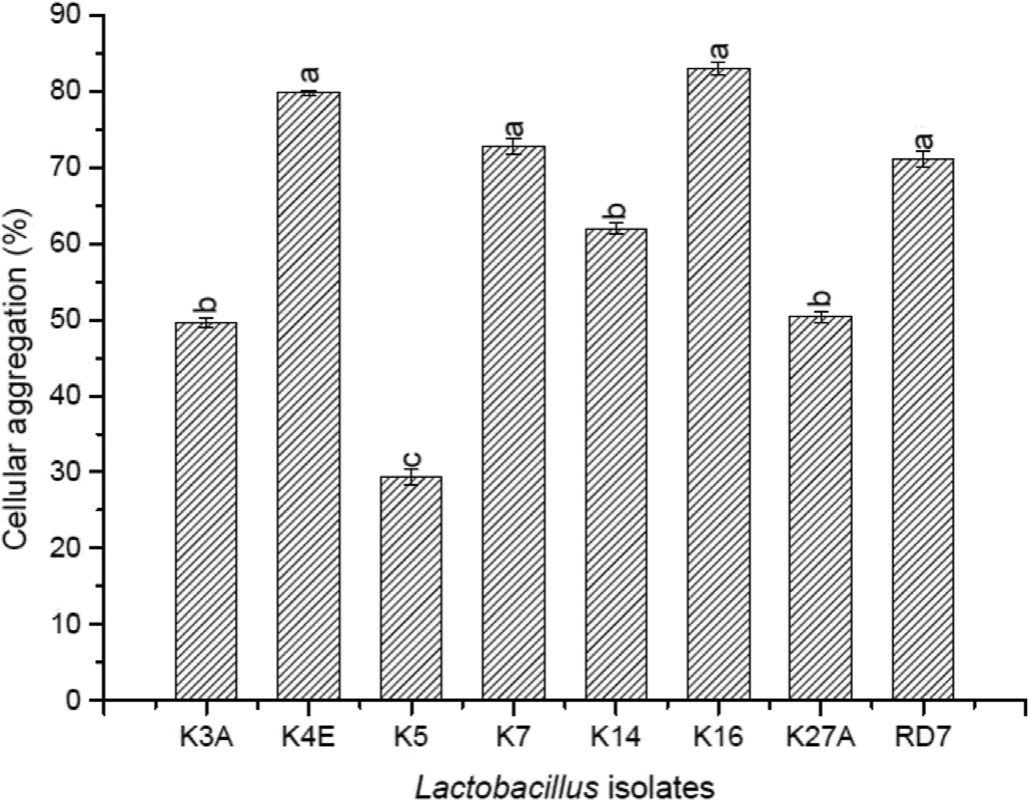
Cellular aggregation of the indigenous *Lactobacillus* isolates. ∗Values are mean ± standard deviation of triplicate determinations (n = 3). Values bearing different superscripts differ significantly (P < 0.05).

The rate of aggregation was determined in percentage for the eight indigenous *Lactobacillus* isolates that differed significantly from each other (Fig. 9). K16 (83 ± 0.86) showed the highest aggregation followed by K4E (79.80 ± 0.35), K7 (72.8 ± 1.05) and RD7 (71 ± 1.05). The lowest aggregating efficacy was reported by K5 (29.4 ± 1.08). All of the eight indigenous *Lactobacillus* isolates showed a significant increase in their co-aggregation efficacy (%) with the six test organisms mentioned above after 0 h, 4 h, and 24 h of incubation (Table 6). Out of all the lactic isolates employed, K7 showed the highest co-aggregation with the test organisms ranging from 54.66 ± 1.37 to 79.83 ± 4.28 followed by K4E showing co-aggregation ranging from 69.52 ± 2.45 to 76.55 ± 2.15. Least co-aggregation was observed in K14 from 28.83 ± 2.22 to 39.16 ± 1.26 after 24 h against the five test organisms used in the study.

**Table 6:** Co-aggregation ability of the indigenous *Lactobacillus* strains against different test organisms.

Strain Combination	Co-aggregation (%)		
RD7 with:	0 h	4 h	24 h
*Salmonella typhi*	0.33 ± 0.034^a^	12.62 ± 2.53^c^	61.0 ± 2.74^i^
*Staphlococcus aureus*	0.50 ± 0.020^a^	14.33 ± 1.65^c^	47.16 ± 1.25^g^
*Bacillus cereus*	0.33 ± 0.028^a^	19.61 ± 2.05^d^	64.66 ± 2.33^i^
*Listeria monocytogenes*	0.16 ± 0.031^a^	15.0 ± 1.43^c^	45.83 ± 1.74^g^
*Escherichia coli*	0.50 ± 0.101^a^	20.83 ± 1.72^d^	69.33 ± 2.81^h^
K4E with:			
*Salmonella typhi*	0.33 ± 0.092^a^	14.83 ± 1.62^c^	69.52 ± 2.45^i^
*Staphlococcus aureus*	0.16 ± 0.118^a^	21.33 ± 1.24^d^	62.50 ± 2.10^i^
*Bacillus cereus*	0.50 ± 0.100^a^	21.50 ± 1.10^d^	65.51 ± 1.59^i^
*Listeria monocytogenes*	0.33 ± 0.065^a^	24.50 ± 1.30^d^	73.00 ± 2.05^h^
*Escherichia coli*	0.16 ± 0.082^a^	26.33 ± 1.05^d^	76.55 ± 2.15^h^
K14 with:			
*Salmonella typhi*	1.66 ± 0.088^b^	4.66 ± 0.297^b^	28.83 ± 2.22^d^
*Staphlococcus aureus*	0.50 ± 0.064^a^	6.16 ± 0.332^b^	31.83 ± 2.80^e^
*Bacillus cereus*	0.83 ± 0.101^b^	15.53 ± 1.07^c^	36.33 ± 1.26^e^
*Listeria monocytogenes*	0.50 ± 0.092^a^	10.66 ± 1.13^c^	38.00 ± 2.04^e^
*Escherichia coli*	1.00 ± 0.055^a^	15.83 ± 1.15^c^	39.16 ± 2.28^e^
K7 with:			
*Salmonella typhi*	0.33 ± 0.020^a^	15.83 ± 1.67^c^	54.66 ± 1.37^f^
*Staphlococcus aureus*	0.66 ± 0.094^b^	20.55 ± 1.35^d^	61.66 ± 2.47^i^
*Bacillus cereus*	0.50 ± 0.065^a^	22.50 ± 2.07^d^	79.83 ± 4.28^h^
*Listeria monocytogenes*	0.83 ± 0.043^b^	16.66 ± 3.40^c^	73.00 ± 1.77^h^
*Escherichia coli*	0.33 ± 0.075^a^	24.51 ± 2.25^d^	71.00 ± 3.04^h^
K16 with:			
*Salmonella typhi*	0.83 ± 0.033^b^	21.33 ± 2.20^d^	49.83 ± 1.84^f^
*Staphlococcus aureus*	0.50 ± 0.065^a^	22.83 ± 3.17^d^	52.66 ± 4.05^f^
*Bacillus cereus*	0.33 ± 0.047^a^	24.16 ± 1.85^d^	52.33 ± 3.58^f^
*Listeria monocytogenes*	0.66 ± 0.053^b^	21.33 ± 2.36^d^	57.66 ± 3.06^f^
*Escherichia coli*	0.50 ± 0.077^a^	17.16 ± 1.18^c^	54.16 ± 2.25^f^
K27A with:			
*Salmonella typhi*	0.83 ± 0.053^b^	12.33 ± 1.14^c^	46.16 ± 1.32^g^
*Staphlococcus aureus*	0.50 ± 0.042^a^	15.16 ± 3.16^c^	42.00 ± 4.13^g^
*Bacillus cereus*	0.83 ± 0.077^b^	20.16 ± 1.11^d^	38.16 ± 2.43^e^
*Listeria monocytogenes*	0.33 ± 0.080^a^	22.83 ± 2.32^d^	50.00 ± 4.11^f^
*Escherichia coli*	1.00 ± 0.092^b^	24.16 ± 1.20^d^	51.66 ± 2.85^f^
K5 with:			
*Salmonella typhi*	1.33 ± 0.062^b^	33.16 ± 1.27^e^	42.83 ± 4.05^g^
*Staphlococcus aureus*	1.00 ± 0.022^b^	30.50 ± 2.09^e^	45.50 ± 3.32^g^
*Bacillus cereus*	0.50 ± 0.038^a^	32.00 ± 4.10^e^	47.55 ± 3.05^g^
*Listeria monocytogenes*	1.33 ± 0.090^b^	29.66 ± 2.36^e^	54.66 ± 2.87^f^
*Escherichia coli*	1.00 ± 0.095^b^	28.16 ± 3.08^d^	52.83 ± 2.45^f^
K3A with:			
*Salmonella typhi*	1.16 ± 0.095^b^	28.83 ± 2.16^d^	54.50 ± 3.57^f^
*Staphlococcus aureus*	1.00 ± 0.082^b^	30.16 ± 1.35^e^	57.33 ± 4.59^f^
*Bacillus cereus*	0.50 ± 0.078^a^	32.33 ± 2.20^e^	48.00 ± 3.15^g^
*Listeria monocytogenes*	1.33 ± 1.01^b^	27.83 ± 1.56^d^	58.66 ± 2.87^f^
*Escherichia coli*	1.00 ± 0.070^b^	33.16 ± 3.08^e^	55.66 ± 4.10^f^

Values are mean ± SD of three independent determinations (n = 3) of each sample. Values bearing different superscripts in each column differ significantly (P < 0.05).

## 4 Discussions

In most of the probiotic microorganisms, the enzymes viz. α-galactosidase and β-glucosidase are found as crude matter and henceforth they show activities towards p-nitrophenyl-α-D-galactopyranoside and p-nitrophenyl-β-D-glucopyranoside (Tochikura et al., 1986). These enzymes functions as an indicator for the liberation of bioactive isoflavones from lactic strains with a promising action of improved hydrolyzation of non-digestible oligosaccharides (Otieno et al., 2006). Previous studies have shown a strain-dependent α-Gal activity in soy milk medium for the development of compact soy curd with less whey separation during the 24 h fermentation period (Hati et al., 2012). With consideration to our study similar findings were reported by Myagmardorj et al. (2018) with an increased α-Gal and β-Glu activity after 24 h of soymilk fermentation. Hati et al. (2012) reported with higher α-Gal activity in soymilk medium (without any nutritional fortification) fermented by *L. rhamnosus* C6 strain. Le Blanc et al. (LeBlanc et al., 2004) reported α-Gal activity at a constant rate with *Lactobacillus* strains in the soymilk medium. In a study reported by Otieno and Shah (2007), the β-glucosidase enzyme units were 0.294 μM/ml for *L*. *casei* 2607, followed by 0.199 μM/ml for *L*. *casei* ASCC 290*,* 0.177 μM/ml for *L*. *acidophilus* 33 200, 0.137 μM/ml for *L*. *acidophilus* 4962, 0.087 μM/ml for *L*. *acidophilus* 4461 which were quite less as compared to the β-glucosidase activity showed by our strains that ranged from 0.122 to 0.409 μM/ml. These results could be explained by the different strains used for screening, as the β-glucosidase activity is strain-dependent (Otieno and Shah, 2007). Hence, for the development of functional foods with higher estrogenicity facilitating bioavailability of active isoflavones, β-glu secretion by lactic acid bacteria is considered as a major parameter [21].

The exopolysaccharide derived from LAB strains along with medium (carbon sources) composition and growth conditions (temperature, pH, etc.) plays a pivotal role in improvising texture, moth feel and total yield for formulations applied in food fermentation industries (Dilna et al., 2015). These EPS producing strains possibly possess greater efficacy in withstanding stresses (Stack et al., 2010) and surviving while passing through the gastrointestinal tract (Lindström et al., 2012). However, our results differ from Cerning et al. (1994) who reported with glucose as a better carbon source for *L. casei* CG11 for production of EPS and lactose as an inefficient source and with Ruas-Madiedo (Ruas-Madiedo and de los Reyes-Gavilán, 2005) who reported 1–10 g of EPS production per liter of growth media used. Whereas, in our results above sucrose proved to be the efficient carbon source which resulted in higher EPS production. There are reports for EPS production from various *Lactobacillus* strains from starter dough for Chinese steamed buns (Luangsakul et al., 2009) and fermented bamboo shoots (Chen et al., 2010).

To be identified as a probiotic, one of the prerequisites is the ability to survive in the gut environment passage (Fernández et al., 2003) with high acidic pH (Table 2), overcoming against 0.5% bile salts (Table 3), gastrointestinal (Table 4a, Table 4ba and 4b) and pancreatic juices (Table 5). The results are in agreement with Argyri et al. (2013) where the viable nature of the *Lactobacillus* strains was maintained when exposed to lower pH values in the range from 2.5-4.0. Bile tolerance is one of the crucial parameters to be analysed for probiotic bacteria since it determines their efficacy for survival in the small intestine (Ruiz et al., 2013) and the suggested concentration of bile salts for probiotics is between the ranges of 0.15–0.5% since this is the physiological concentration range which is being met in the gastrointestinal tract (Papadimitriou et al., 2015). Furthermore, microbial bile salt hydrolase functions in detoxifying bile salts help in increasing the prolonged survival in the intestine followed by persistence of the strains and possibly the profitable impacts related to it (Begley et al., 2006).

Probiotic lactobacilli organisms are generally considered rich in proteolytic activity due to the presence of aminopeptidases by hydrolysing peptides in the growth medium (Hati et al., 2015). The addition of lactic strains to soymilk resulted in the release of free amino acid content and to support our study, a similar observation was reported by Rekha and Vijayalakshmi (2008) in soy fermentation with various lactobacilli after 24 h incubation. Donkor et al. (2007), reported that the extent of proteolysis differed among the lactic strains and appeared to be time dependant. The findings concluded that the amount of released amino groups and peptides increased slightly till 0.80 nm during fermentation from 0 to 12 h for few strains viz. *L. acidophilus* L10, *L. acidophilus* La 4962, *B. lactis* B94, *B. longum* Bl 536, *L*. *casei* L26, and *L*. *casei* Lc 279) but increased significantly (P < 0.05) for all strains from 12 to 24 h till 1.80 nm.

For ensuring safety and non-virulence mode of probiotic microorganisms, antibiotic susceptibility was carried out. The resisting nature of Lactobacilli to kanamycin as reported earlier for members of the genus *Lactobacillus* stands with similarity to our observations (Karapetkov et al., 2011).

Lactobacilli are generally sensitive towards inhibitors for cell wall synthesis and stand resistant to various aminoglycosides (vancomycin, gentamycin kanamycin, streptomycin) since due to the absence of cytochrome-mediated electron transport enabling antibiotic uptake (Mayrhofer et al., 2010). Similarly due to the presence of D-Ala-D-lactate in the peptidoglycan of Lactobacilli makes it resistant to vancomycin which is an intrinsic widespread phenomenon (Delgado et al., 2007). Hence, the resistance to kanamycin and vancomycin in the study does not possess any risk of antibiotic-resistance genes transfer.

Few lactobacilli seem to be intrinsically resistant (Mandras et al., 2016) to second-generation quinolones-fluoroquinolones, viz. norfloxacin, ciprofloxacin, levofloxacin by a presently unknown resistance mechanism (Hummel et al., 2007). Hummel et al. (2007) investigated if point mutations in the gyrA or parC genes are responsible for fluoroquinolone resistance in lactic acid bacteria. The genetic basis for the resistance could not be verified since no mutations typical of quinolone resistance were detected in the quinolone determining regions of the parC and gyrA genes. Enzymatic inactivation such as for aminoglycosides viz. vancomycin, kanamycin, streptomycin or quinolones viz. ciprofloxacin, norfloxacin, nalidixic acid restricts the binding of these antibiotics with their specific targets, as reported for *Lactobacillus* and *Enterococcus* for the 16S rRNA of the 30S ribosomal bacterial subunit and DNA gyrase, respectively, that explains the intrinsic resistance to both groups of antibiotics (aminoglycosides and quinolones) (Clementi and Aquilanti, 2011, Jaimee and Halami, 2016, Álvarez-Cisneros et al., 2019). Single nucleotide polymorphisms (SNPs) could also be the reason behind causing resistance against the synthetic drugs viz. quinolones, sulfonamides, and trimethoprim (Ruiz, 2003) and mutations within the rpsL gene that encodes the ribosomal protein S12, which may led to a high-level streptomycin resistance (Nair et al., 1993).

Osuntoki et al. (2008) in a study reported the antibacterial activity of *Lactobacillus* spp. from fermented dairy foods against Enterotoxigenic *E. coli* (4.2 mm), *Salmonella typhimurium* (4.3 mm) and *Listeria monocytogenes* (5.0 mm). The *Lactobacillus* strains employed in our study have shown much better antimicrobial capability as presented in Fig. 5. Similarly, Gautam et al. (2014) studied the antagonism of lactic acid bacteria isolated from Dulliachar-a salted pickle (traditional food from North-eastern India) and was found to produce broad-spectrum antibacterial activity against foodborne pathogens viz*. L. monocytogenes, S. aureus* and *B. cereus*.

The indigenous *Lactobacillus* cultures employed in the study can be claimed as potential antioxidant suppressors as they managed to scavenge the ABTS^+^ thereby reducing the ferryl myoglobin radical after a period of 24 h. Rjiniemon et al. (2015) also reported antioxidative activities from LAB isolated from fermented foods that could be employed for the treatment of chronic diseases (cancer, diabetes).

As per the reports, fermented foods infused with LAB minimizes the level of cholesterol as such in traditional foods viz. *tempeh*, fermented soybean foods and *kefir* (Hermosilla et al., 1993). *Lactobacillus* isolates used in the study could assimilate cholesterol as depicted in Fig. 7 and based on the results we can presume that it may likely do a similar activity in the human gut too thereby reducing the dietary cholesterol. With contrast to the reports stated by Ziarno (2007), *L. rhamnosus* strain showed cholesterol assimilation ranging from 13.6% to 17.5% and Kathiriya et al. (2018) reported significant cholesterol reduction (3.36%) by *L. rhamnosus* NS6 which was quite lower than that reported by our indigenous lactic strains.

To study the attainable adherence of the *Lactobacillus* isolates to the intestinal mucus, cell surface hydrophobicity was carried out. Adherence was denoted by adhering capability to n-hexadecane (alkane hydrocarbon). The indigenous lactic strains employed in our study showed better-adhering results than the *Lactobacillus* strains (Samot et al., 2011). Furthermore, in agreement with our study, Del Re et al. (Del Re et al., 2000) reported that strains with higher hydrophobic surface possess a higher capacity to adhere to intestinal epithelial cells and solid materials as well.

Aggregation between microorganisms belonging to similar strain (auto-aggregation) or between strains that genetically differ (co-aggregation) is of considerable importance for preliminary probiotic screening (Jankovic et al., 2003). In a study conducted by Reid et al. (1988), it was reported that the co-aggregating parameter of *Lactobacillus* strains with uropathogens is a primary factor for the maintenance of healthy urogenital microflora. Previous reports have suggested that the cellular aggregation by *Lactobacillu*s strains is protein-mediated and contrastingly, others have reported lipoteichoic acids mediation (Kos et al., 2003). Efficient cellular aggregation and co-aggregation to intestinal mucosa could result in the proper proliferation and maintenance of probiotic bacteria in the gastrointestinal tract (Servin and Coconnier, 2003).

## 5 Conclusion

The study has provided valuable information on the *in vitro* characteristics of the indigenous *Lactobacillus* isolates from the ethnic fermented foods of Meghalaya. This has helped in the identification of potential probiotic candidates that can be used for further investigation for clinical trials and elucidate their probiotic potential to be used as starter cultures and development of novel functional fermented foods.

## Authorship

Sujit Das: Conceptualization, Methodology, Validation, Formal Analysis, Investigation, Writing – Original Draft, Writing – Reviewing & Editing.

Birendra Kumar Mishra: Supervision, Writing – Reviewing & Editing.

Subrota Hati: Supervision, Writing – Reviewing & Editing.

## Declaration of Competing Interest

None of the authors has any conflicts of interest to declare.
